# Supplementary material for: Diabetic foot ulcers: Retrospective comparative analysis from Sicily between two eras
Source: PLoS One. 2021 Dec 7;16(12):e0259405. doi: 10.1371/journal.pone.0259405 (PMC8651101; doi:10.1371/journal.pone.0259405)
Supplement: S1 Table — (DOC) [file pone.0259405.s001.doc]

**Table S1**

|  | **2008-2013**  **(n=149)** | |  | **2014-2019**  **(n=181)** | |  |
| --- | --- | --- | --- | --- | --- | --- |
|  | **Alive**  **(n= 83)** | **Dead**  **(n=66)** | ***p^a^*** | **Alive**  **(n= 137)** | **Dead**  **(n=44)** | ***p^b^*** |
| **Gender**  Males  Females | 58 (69.9%)  25 (30.1%) | 39 (59.1%)  27 (40.9%) | 0.115 | 93 (67.9%)  44 (32.1%) | 29 (65.9%)  15 (34.1%) | 0.472 |
| **Arterial hypertension** | 72 (86.7%) | 63 (95.5%) | 0.061 | 106 (77.4%) | 41 (93.2%) | 0.013 |
| **Dyslipidemia** | 70 (84.3%) | 57 (86.4%) | 0.457 | 89 (65%) | 31 (70.5%) | 0.316 |
| **Cardiovascular disease**  Myocardial infarction  Stroke  Cardiac insufficiency | 25 (30.1%)  0  0 | 28 (42.4%)  3 (4.5%)  0 | 0.083  0.085 | 33 (24.1%)  13 (9.5%)  0 | 19 (43.2%)  4 (9.1%)  1 (2.3%) | 0.014  0.602  0.075 |
| **Smoking**  Current  Former | 19 (22.9%)  2 (2.4%) | 15 (22.7%)  2 (3%) | 0.973  0.599 | 40 (29.2%)  41 (29.9%) | 10 (22.7%)  12 (27.3%) | 0.419  0.447 |
| **Chronic kidney disease**  Mild  Moderate  Severe | 29 (34.9%)  13 (15.7%)  1 (1.2%) | 23 (34.8%)  21 (31.8%)  10 (15.2%) | 0.565  0.016  0.001 | 32 (23.4%)  30 (21.9%)  8 (5.8%) | 10 (22.7%)  12 (27.3%)  6 (13.6%) | 0.555  0.294  0.091 |
| **SIRS** | 10 (12%) | 14 (21.2%) | 0.099 | 22 (16.1%) | 8 (18.2%) | 0.451 |
| **Peripheral vascular disease** | 29 (34.9%) | 38 (57.6%) | 0.005 | 82 (59.9%) | 31 (70.5%) | 0.139 |
| **Retinopathy**  Mild non-proliferative  Moderate non-proliferative  Proliferative | 22 (26.5%)  7 (8.4%)  0 | 14 (21.2%)  10 (15.2%)  10 (9%) | 0.290  0.154 | 11 (8%)  3 (2.2%)  25 (18.2%) | 1 (2.3%)  0  0 | 0.162  0.431  0.325 |
| **Hypolipidemic therapy** | 69 (83.1%) | 58 (87.9%) | 0.283 | 97 (70.8%) | 33 (75%) | 0.370 |
| **Antiplatelet therapy** | 69 (83.1%) | 58 (87.9%) | 0.283 | 119 (86.9%) | 43 (97.7%) | 0.029 |
| **Diabetic treatment**  Oral hypoglycaemic agents  Basal-bolus insulin  Oral hypoglycaemic agents + long-acting insulin | 20 (24.1%)  38 (45.8%)  25 (30.1%) | 8 (12.1%)  42 (63.6%)  16 (24.2%) | 0.048  0.022  0.271 | 12 (8.8%)  98 (71.5%)  27 (19.7%) | 4 (9.1%)  36 (81.8%)  4 (9.1%) | 0.577  0.122  0.077 |
| **Lesion type**  Hischaemic  Neuropathic  Neurohischaemic | 3 (3.6%)  36 (43.4%)  44 (53%) | 4 (6.1%)  18 (27.3%)  43 (65.2%) | 0.374  0.031  0.092 | 14 (10.2%)  46 (33.6%)  77 (56.2%) | 8 (18.2%)  6 (13.6%)  30 (68.2%) | 0.128  0.007  0.109 |
| **Affected foot**  Right  Left  Both | 34 (41%)  43 (51.8%)  6 (7.2%) | 33 (50%)  24 (36.4%)  9 (13.6%) | 0.175  0.061  0.155 | 70 (51.1%)  55 (40.1%)  11 (8%) | 21 (47.7%)  16 (36.4%)  7 (15.9%) | 0.415  0.396  0.112 |
| **Lesion area**  I toe  Distal extremities  Lateral plantar  Medial plantar  Calcanear  Dorsal | 12 (14.5%)  11 (13.3%)  40 (48.2%)  33 (39.8%)  5 (6%)  0 | 9 (13.6%)  14 (21.2%)  32 (48.5%)  26 (39.4%)  1 (1.5%)  2 (3%) | 0.540  0.142  0.551  0.550  0.167  0.195 | 15 (10.9%)  15 (10.9%)  73 (53.3%)  48 (35%)  11 (8%)  11 (8%) | 5 (11.4%)  3 (6.8%)  28 (63.6%)  12 (27.3%)  5 (11.4%)  4 (9.1%) | 0.564  0.319  0.152  0.223  0.341  0.518 |
| **Osteomyelitis** | 22 (26.5%) | 3 (4.5%) | 0.393 | 31 (22.6%) | 2 (4.5%) | 0.136 |
| **Revascularization treatment** | 22 (26.5%) | 26 (39.4%) | 0.068 | 31 (22.6%) | 9 (20.5%) | 0.471 |
| **Surgery treatment**  Minor amputation  Major amputation | 17 (20.5%)  1 (1.2%) | 15 (22.7%)  3 (4.5%) | 0.425  0.224 | 43 (31.4%)  5 (3.6%) | 15 (34.1%)  3 (6.8%) | 0.536  0.354 |
| **VAC therapy** | 19 (22.9%) | 21 (31.8%) | 0.150 | 44 (32.1%) | 15 (34.1%) | 0.472 |
| **Stage**  **A**  **B**  **C**  **D** | 6 (7.2%)  37 (44.6%)  3 (3.6%)  40 (48.1%) | 0  21 (31.8%)  2 (3%)  40 (60.6%) | 0.028  0.078  0.607  0.129 | 2 (1.5%)  69 (50.4%)  1 (0.7%)  65 (47.4%) | 2 (4.5%)  17 (38.6%)  1 (2.3%)  24 (54.5%) | 0.147  0.118  0.428  0.259 |
| **Grade**  0  1  2  3 | 1 (1.2%)  30 (36%)  43 (51.8%)  5 (6%) | 0  25 (37.8%)  41 (62.1%)  4 (6.1 %) | 0.556  0.831  0.137  0.772 | 1 (0.7%)  55 (40.1%)  68 (49.6%)  18 (13.1%) | 0  17 (38.6%)  25 (56.8%)  7 (15.9%) | 0.897  0.502  0.546  0.404 |

p^a^ comparison between alive and dead patients in the period 2008-2013

p^b^ comparison between alive and dead patients in the period 2014-2019
