# Supplementary material for: Diabetic foot ulcers: Retrospective comparative analysis from Sicily between two eras
Source: PLoS One. 2021 Dec 7;16(12):e0259405. doi: 10.1371/journal.pone.0259405 (PMC8651101; doi:10.1371/journal.pone.0259405)
Supplement: S2 Table — (DOCX) [file pone.0259405.s002.docx]

**Table S2.**

|  | **Patients hospitalized**  **2008-2013** | | |  | **Patients hospitalized**  **2014-2019** | | |  |  |
| --- | --- | --- | --- | --- | --- | --- | --- | --- | --- |
|  | **All**  **(n=149)** | **Alive**  **(n=83)** | **Dead**  **(n=66)** |  | **All**  **(n=181)** | **Alive**  **(n=137)** | **Dead**  **(n=44)** |  |  |
|  | ***Mean ± SD*** | ***Mean ± SD*** | ***Mean ± SD*** | ***p^a^*** | ***Mean ± SD*** | ***Mean ± SD*** | ***Mean ± SD*** | ***p^b^*** | ***p^c^*** |
| ***General***  ***Parameters*** |  |  |  |  |  |  |  |  |  |
| Age at onset of diabetic foot (years) | 64.9 ± 11.7 | 61.8 ± 11.7 | 68.9 ± 10.3 | <0.001 | 65.6 ± 12.5 | 63.4 ± 12.2 | 72.5 ± 10.9 | <0.001 | 0.620 |
| BMI (Kg/m^2^) | 29.2 ± 4.75 | 29.2 ± 5.1 | 29.3 ± 11.7 | 0.812 | 29.1 ± 4.52 | 29.3 ± 4.3 | 28.6 ± 4.91 | 0.395 | 0.809 |
| Duration of diabetes (years) | 18.2 ± 11.1 | 16.3 ± 9.6 | 20.5 ± 12.2 | 0.002 | 20.2 ± 12.5 | 19.2 ± 12.6 | 23.3 ± 13.8 | 0.060 | 0.125 |
| Healing time (days) | 24.4 ± 12.1 | 22.6 ± 10.5 | 26.6 ± 13.5 | 0.051 | 32.9 ± 23.4 | 33.9 ± 22.4 | 31.4 ± 26.8 | 0.670 | <0.001 |
| ***Metabolic parameters*** |  |  |  |  |  |  |  |  |  |
| Creatinine (mg/dL) | 1.06 ± 0.49 | 0.92 ± 0.34 | 1.24 ± 0.59 | <0.001 | 1.35 ± 1.09 | 1.16 ± 0.84 | 1.94 ± 1.51 | <0.001 | 0.003 |
| eGFR (mL/min) | 76.1 ± 27.8 | 86.2 ± 25.2 | 63.5 ± 25.9 | <0.001 | 71.5 ± 33.2 | 77.6 ± 31.3 | 52.6 ± 32.1 | <0.001 | 0.176 |
| Urinary albumin (g/24h) | 0.25 ± 0.48 | 0.21 ± 0.41 | 0.31 ± 0.55 | 0.154 | 0.41 ± 0.65 | 0.37 ± 0.64 | 0.52 ± 0.69 | 0.187 | 0.018 |
| HbA1c (%) | 9.98 ± 1.78 | 10.1 ± 1.84 | 9.88 ± 1.71 | 0.549 | 10.1 ± 2.09 | 10.1 ± 2.12 | 9.76 ± 1.34 | 0.358 | 0.873 |
| Total cholesterol (mmol/L) | 4.14 ± 1.07 | 4.31 ± 1.09 | 3.93 ± 1.02 | 0.033 | 3.52 ± 0.92 | 3.54 ± 0.91 | 3.46 ± 11.7 | 0.638 | <0.001 |
| HDL cholesterol (mmol/L) | 0.82 ± 0.28 | 1.02 ± 0.31 | 0.92 ± 0.28 | 0.046 | 0.87 ± 0.28 | 0.82 ± 0.26 | 0.81 ± 0.31 | 0.809 | <0.001 |
| LDL cholesterol (mmol/L) | 2.41 ± 0.95 | 2.55 ± 0.98 | 2.22 ± 0.89 | 0.037 | 2.01 ± 0.77 | 2.01 ± 0.73 | 1.94 ± 0.89 | 0.608 | <0.001 |
| Triglycerides (mmol/L) | 1.64 ± 0.72 | 1.61 ± 0.74 | 1.70 ± 0.69 | 0.407 | 1.53 ± 0.65 | 1.53 ± 0.64 | 1.54 ± 0.71 | 0.921 | 0.136 |
| ***Inflammatory parameters*** |  |  |  |  |  |  |  |  |  |
| VES (mm) | 42.2 ± 23.8 | 39.1 ± 21.6 | 46.2 ± 25.9 | 0.072 | 51.1 ± 24.3 | 50.6 ± 24.1 | 52.5 ± 25.1 | 0.648 | 0.001 |
| PCR (mg/L) | 56.9 ± 39.5 | 54.4 ± 38.6 | 60.2 ± 40.8 | 0.380 | 57.6 ± 65.9 | 59.1 ± 66.5 | 53.2 ± 44.7 | 0.608 | 0.916 |

p^a^ comparison between dead and alive patients hospitalized in the period 2008-2013

p^b^ comparison between dead and alive patient hospitalized in the period 2014-2019

p^c^ comparison between all patients hospitalized in the periods 2008-2013 and 2014-2019
