# Supplementary material for: Diabetic foot ulcers: Retrospective comparative analysis from Sicily between two eras
Source: PLoS One. 2021 Dec 7;16(12):e0259405. doi: 10.1371/journal.pone.0259405 (PMC8651101; doi:10.1371/journal.pone.0259405)
Supplement: Attachment — Submitted filename: response to reviewers.docx [file pone.0259405.s003.docx]

**Comments to the Author**

Reviewer #1: In the present article entitled “Diabetic Foot Ulcers: a retrospective analysis of Sicilian patients hospitalized in 2008-
2013 and 2014-2019 years” Giordano and colleagues, have retrospectively analyzed incidence, management and
mortality of DFU in Sicilian Type 2 diabetic patients. Although the manuscript contains extensive amount of work, I have several concerns about data presentation and interpretation. Here are the specific comments.

Title is should be in lines with the overall objective of the study; think title may be changed to 'DFU: improvement in outcomes after implementing standardized care' or 'DFU: retrospective comparative analysis from Sicily between two eras'

Thanks for this interesting suggestion. We changed the title in “Diabetic Foot Ulcers: retrospective comparative analysis from Sicily between two eras”

Abstract:

Aim: 'lustra', can use standard words like 'era'

Thanks for the comment. We changed lustra with era.

Methods: can change to: we compared the two eras, era1: 2008-13, era2: 2014-19. In era 1, n=149, and so on...

Thanks for this suggestion. We changed the sentence.

Conclusion: advanced age/senility itself is a principal risk factor of death. What was done to remove this bias?

Thanks for the comment. We did not remove this bias, because we calculated how much the age at onset of diabetic foot ulcer influence on the risk of death, calculating the odds ratio.


Fig 1 A, B, specify unit on x axis in fig 1, a and b

Thanks for the comment. We added the units in the figure 1.

table 1, typo error: Ischemic, not Hischemic

Thanks for the comment. We corrected the typing error.

Methodologies: Ref check: ref 5: International Working Group guidelines on the Diabetic Foot is not same as NIHCE guidelines.: needs reference check.
Reference check again required: ref 26 defines 'Percutaneous transluminal angioplasty and stenting for carotid artery stenosis', the authors may choose to instead choose criteria for Peripheral / femoral angioplasty since we are looking at DFU.

Thanks for the comment. We checked and corrected the references.


Statistically: it will be more logical to compared dead patients from both eras to arrive at any conclusion regarding effectiveness of changed guidelines other than decreased mortality rates.

Thanks for the interesting suggestion. We compared patients who died in the first era with second era.

Overall, the supplementary data needs to be added for patient details and especially the methodological inconsistencies need to be addressed.

Thanks for the comment. We added supplemental tables.

Reviewer #2: Ref: PONE-D-21-21103

In the present article entitled “Diabetic Foot Ulcers: a retrospective analysis of Sicilian patients hospitalized in 2008-2013 and 2014-2019 years” Guarnotta et al. have retrospectively analyzed incidence of DFU in Sicilian population. Further they have attempted to look at the change in mortality between two time periods. The study design is simple and the results are reasonably by the data. However, some points need to be addressed to make the study robust for the publication.
Major concern: the conclusion does not clearly answer the aim of the study.

line 284: Conclusion: "MDFT could be considered promising in the future for more rapid auditing of diabetic foot ulcer numbers and for establishment of predictive risk factors causing mortality" How do the findings of this study clearly establish the role of a multi-disciplinary team, what exact quality improvement was achieved by such implementation needs to be addressed.

Thanks for the comment. We added in the conclusion section that multidisciplinary team have had an impact on reducing mortality due to a better management of diabetic foot.

Statistical analysis.to reach any conclusions, the patients who died from era I need to be compared to era II
Thanks for the comment. We compared patients who died in the two eras (Tables 4 and 5).

Minor conclusion:
Authors need to address the "latent period of diabetes before patients reach the clinic"

Thanks for the comment. We changed it.

Title can be improved. “...2008-2013 and 2014-2019 years” needs to be rephrased.

Thanks we changed the title as suggested.
Authors have used “Lustra”. Please stick to use of standard English words.

Thanks for the suggestion, we changed lustra with eras.

methods: line 94: International Working Group guidelines on the Diabetic Foot is may not be same as NIHCE guidelines.: needs reference check/ justify

Thanks for the suggestion, We corrected the reference.

line 187: "who dies higher frequency" can be changed to "that died had". Please fix.

Thanks for the comment. We modified the sentence.

"figure 1: specify unit of time on x axis"

Thanks for the suggestion. We specified the units in the figure.

"table 5, typo error: last line: hospitalized in the patients"

Thanks for the comment. We checked and corrected the typing error.

overall: use standard abbreviation for number, 'n'

Thanks for the comment. We used standard abbreviations.
